# Testing the Foreign Language Effect on Cognitive Reflection in Older Adults

**DOI:** 10.3390/brainsci11111527

**Published:** 2021-11-18

**Authors:** Mariana Vega-Mendoza, Patrik Hansson, Daniel Eriksson Sörman, Jessica K. Ljungberg

**Affiliations:** 1 Department of Health, Education and Technology, Engineering Psychology, Luleå University of Technology, 971 87 Luleå, Sweden; daniel.sorman@ltu.se (D.E.S.); jessica.korning-ljungberg@ltu.se (J.K.L.); 2 Department of Psychology, Umeå University, 907 36 Umeå, Sweden; patrik.hansson@umu.se

**Keywords:** foreign language effect, bilingualism, multilingualism, aging, older adulthood, decision-making, reasoning

## Abstract

An increasing number of people around the world communicate in more than one language, resulting in them having to make decisions in a foreign language on a daily basis. Interestingly, a burgeoning body of literature suggests that people’s decision-making is affected by whether they are reasoning in their native language (NL) or their foreign language (FL). According to the foreign language effect (FLe), people are less susceptible to bias in many decision-making tasks and more likely to display utilitarian cost-benefit analysis in moral decision-making when reasoning in a FL. While these differences have often been attributed to a reduced emotionality in the FL, an emerging body of literature has started to test the extent to which these could be attributable to increased deliberation in the FL. The present study tests whether increased deliberation leads to a FLe on cognitive reflection in a population of older adults (*M*_age_ = 65.1), from the successful aging project in Umeå, Sweden. We explored whether performance on a 6-item version of the cognitive reflection test (CRT) adapted to Swedish would differ between participants for whom Swedish was their NL and those for whom Swedish was their FL. The CRT is a task designed to elicit an incorrect, intuitive answer. In order to override the intuitive answer, one requires engaging in deliberative, analytical thinking to determine the correct answer. Therefore, we hypothesized that if thinking in a FL increases deliberation, then those performing the task in their FL would exhibit higher accuracy rates than those performing in their NL. Our results showed that age and level of education predicted performance on the task but performance on the CRT did not differ between the NL and the FL groups. In addition, in the FL group, proficiency in the FL was not related to performance in the CRT. Our results, therefore, do not provide evidence that thinking in a FL increases deliberation in a group of older adults performing a logical reasoning task that is not typically associated with an emotional connotation.

## 1. Introduction

Bilingualism and multilingualism are very common and established phenomena all over the world. For example, Singapore has four official languages and the country has adopted a bilingual education policy [1], India specifies 22 languages in the Eighth Schedule of the Indian Constitution [2], and in the European Union, nearly two-thirds of working-age adults report knowledge of at least one foreign language (European Commission, 2019).

Bi- and multilingualism are also complex phenomena. There are many reasons why people in the world communicate in more than one language. In some cases, bi- and multilinguals are speakers of a minority indigenous language who learn the dominant state language. In others, we find instances of so-called neighbourhood multilingualism (e.g., India; [3]). However, a great deal of interest has recently been paid to cases of sequential bi- and multilingualism. Multilinguals can be immigrants who speak their first language(s) as well as the language(s) of their host countries. Or can simply be people who acquired an ability to speak languages further than their native one to gain a competitive edge.

The fact that people around the world are using, learning, and communicating in more than one language has important implications, such as many people making decisions in a language that is not their native tongue on a daily basis. The question arises whether there are differences in the way people make decisions depending on whether they are presented with their native or their foreign language. A burgeoning body of literature suggests so. It has been reported that making decisions in a native language differs from making decisions in a foreign language in some key aspects, a phenomenon known as the foreign language effect (henceforth FLe; [4,5]).

The underlying theoretical basis of the FLe comes largely from the literature on thinking and decision-making, in particular from the influential dual-process framework [6,7]. While different characterizations of the framework are on offer (cf. [8]), they typically pose that two cognitive systems are implicated in reasoning. System 1 is intuitive and affective, whilst System 2 is analytic and rule-governed [6] and the latter is thus often referred to as more ‘rational’. With regards to decision-making, it is generally claimed that engagement of System 2 leads to more deliberative thinking and therefore to better choices. However, under certain circumstances, people are prone to ‘errors’ in judgement and decision-making (i.e., *cognitive biases*) believed to arise from engagement in fast, automatic responses of System 1 (but cf. [9,10] for a proposal on how sometimes adaptive behaviours might come from fast, intuitive reasoning).

For individuals who speak more than one language, it has been reported that certain types of cognitive biases are reduced when presented with decision-making problems in their foreign language (FL) compared with their native language (NL) (for a review see [11]). For example, Keysar et al. [4] used different decision-making paradigms involving risk and loss aversion in a series of six experiments. Results showed that those participants presented with problems in their NL tended to choose more risky options when a problem with the same outcome was framed in terms of losses compared to when it was presented in terms of gains (known as a ‘framing effect’). Conversely, such ‘framing effect’ was not observed (i.e., there was no asymmetry in risk-seeking choice whether presented in terms of losses or gains) when the problem was presented in the FL. The authors proposed an account whereby reasoning in the FL elicits a reduced emotional reaction than reasoning in the NL, which in turn reduces cognitive biases influenced by emotional reactions.

Expanding on Keysar et al. [4]’s findings, Costa et al. [5] tested the generalizability and boundaries of the FLe. To this purpose, they employed a large set of paradigms that either invoked or did not invoke an emotional component. Their first set of results replicated those of Keysar et al. [4]’s such that they reported a reduced framing effect for loss aversion biases in the FL compared with the NL. Moreover, the FLe was observed in other types of problems too, in particular in paradigms related to mental accounting and most of the risk aversion problems scenarios (since then, framing effects and/or loss aversion biases in the context of FLe have been also explored in other populations of young adults e.g., [12,13,14,15]). Finally, among the paradigms not expected to carry an emotional component, they tested the FLe on logical reasoning using the cognitive reflection test (CRT; [16]). The CRT is a three-item test (in its original version but see methods for a variant with more items) designed to elicit an incorrect, ‘intuitive’ answer (System 1). In order to override the intuitive answer, one requires to engage in deliberative, analytical, and logical thinking (System 2) to determine the correct answer. Costa et al. [5] reported that both the number of correctly answered questions and the number of intuitive wrong answers was similar for people performing the test in their NL and those performing in their FL, thus revealing an absence of FLe in this task. Taken together, results of this study concluded that the FLe seems to generalize to other decision-making paradigms involving emotional components but not to emotionally neutral paradigms (e.g., CRT), thus giving an indication of the scope and potential boundaries of the FLe.

Furthering on the generalizability of the FLe, Costa et al. [17] assessed the FLe in relation to moral decision-making. They presented a series of moral dilemmas to participants in either their NL or their FL in various populations of L2-late learners of different languages. The moral dilemmas included the well-known trolley problem in which participants are asked whether they would sacrifice one individual (by pressing a switch) in order to save five people. They also employed the footbridge dilemma, which asks whether one would sacrifice an individual by pushing him off a bridge in order to save five people. The footbridge dilemma was considered ‘more emotional’ insofar as it entails pushing a man directly, whereas the trolley dilemma was considered less emotional. Under a utilitarian view, the ‘*rational*’ answer is considered the one that achieves the greatest benefit for the greatest number (i.e., answering yes in the examples above). Results of this study showed that for the trolley (or switch) problem, the one considered less emotional, participants chose the utilitarian option most of the time, in both the NL and FL conditions. Conversely, for the footbridge problem, the one considered more emotional, participants presented with the dilemma in their FL made more utilitarian choices than those presented with their NL (but see also related studies on different populations by [18,19]). In addition, although the focus of Costa et al. [17]’s study was on morality, a subset of participants underwent the CRT as well, which was used in that study as a background measure to support the claim that the FL group had good understanding of the problems. In contrast to previous findings (cf. [5]), they reported better performance for participants doing the task in their FL compared to those in their NL. Taken together, their results were interpreted as support for the reduced emotionality in the FL. 

As we have seen, one of the most prominent accounts for the potential underpinnings of the FLe has appealed to reduced emotionality when reasoning in a FL. Such emotional distance in the FL might stem from factors such as the learning modality, for example, classroom instruction for the FL [20]. Using different methodologies such as behavioural, physiological, and neurophysiological measures, researchers have shown differences in emotional processing between the native and the foreign language. For example, taboo or swear words tend to be rated as less strong in the second than in the first language [21]. Caldwell-Harris and Ayçiçeǧi-Dinn ([22]; Experiment 1) showed reduced skin conductance responses to auditorily presented emotional phrases, such as childhood reprimands, when they were heard in the FL compared with the NL, thus suggesting a stronger emotional resonance in the NL than in the FL. In addition, using event-related potentials (ERPs), Opitz and Degner [23] reported a longer latency of an ERP component (Early Posterior Negativity, EPN) when participants read positively (e.g., angel) and negatively (e.g., crime) emotionally loaded words compared to neutral words (e.g., bottle) in the second language (L2) compared to the first language (L1). In sum, the reduced emotionality account has been further supported by evidence that the FL tends to elicit less strong emotional responses than the NL (for a review see [24]).

However, the reduced emotionality account has also been challenged on empirical and theoretical grounds. For example, in the context of moral decision making it has been argued that reduced emotionality cannot be the sole underlying mechanism to explain the FLe. In particular, a study by Geipel et al. [19] found a FLe when participants were faced with what is generally regarded as a more emotional dilemma (i.e., the footbridge problem) but not when they were faced with what is usually considered to be a less emotional moral dilemma (i.e., the trolley problem), consistent with previous findings. Yet, an important element of their study was that the authors also tested the purported reduced emotionality account by gathering self-ratings of emotionality, and in particular ratings of distress after participants completed the moral judgement questions. The reasoning was that if reduced emotionality modulated the FLe, one would then expect to find reduced emotionality ratings in the FL compared with the NL, but only in the case of the more emotionally salient footbridge problem. However, this was not the case, since the authors found reduced emotionality ratings in the FL in both the trolley and the footbridge problem, thus indicating that reduced emotionality is unlikely to modulate the FLe on moral judgment. While these results challenge the reduced emotionality account, the authors also provided an alternative explanation of FLe by pointing to the moderating impact of norm violations. In particular, in another experiment, the authors showed that the FL only influenced moral judgement when the action in question involved a social or moral norm.

Yet, an alternative account that has recently garnered a great deal of attention posits that reasoning in a FL promotes deliberative thinking and explains the FLe in certain cognitive biases (see e.g., [25]). The logic behind this account stems from the proposal that reduced cognitive fluency improves performance on tasks that require more careful processing by making people more cautious of their responses. In other words, a decrease in processing fluency prompts people to slow down and think more carefully about the decision-making situation. Evidence of this can be found in earlier behavioural observations from the judgement and decision-making literature. For example, Alter et al. ([26]; Experiment 1) showed that factors that reduced cognitive fluency such as presenting the CRT items in difficult to read text, led to higher accurate rates than administering the test in easy to read fonts, thus concluding that cognitive disfluency prompted engagement in System 2 (analytic reasoning). Moreover, reduced cognitive fluency created by presenting information in a difficult to read font has also been shown to reduce the number of erroneous responses on distorted questions [27] and to reduce confirmation bias [28]. With that in mind, the ability of disfluency to prompt additional processing and reduce tendency to bias and errors has been well-documented in a variety of relevant contexts (but see [29] for contrasting evidence). In the context of the FLe, it is proposed that thinking in a FL incurs higher processing costs thus reducing cognitive fluency. In turn, cognitive disfluency increases deliberation (see also [11]).

Hayakawa et al. [30] set out to test between the two aforementioned accounts, namely, reduced-emotionality and increased-deliberation. They used a ‘process-dissociation task’, a method that allows one to partial out the effects of deontological responding (related to emotionality) from those of utilitarian responding (related to deliberative reasoning). Results of a series of six experiments focusing on moral dilemmas showed that using a FL reduced deontological responding. In addition, they found no evidence that FL increased utilitarian responding (cf. [17]). They concluded that the moral FLe (MFLe) arises from reduced emotionality rather than increased deliberation.

To sum up, a body of literature has shown that using a FL reduces certain types of cognitive biases and increases utilitarian choices, particularly in paradigms that elicit an emotional component. Our understanding of the FLe is still far from conclusive (for discussions and future directions see [11,31]) and even more, recent discussions argue that indeed the reduced emotionality and the increased deliberation proposals are not mutually exclusive [11,25]. In addition, among the questions that remain open are to what extent factors surrounding the bilingual experience impact the FLe [11]. Most of the studies in the FLe literature have been conducted in populations of young adults, acquiring their FL later in life usually in late childhood or adolescence (e.g., [4,5,18,25,30]). But would the FLe still be observed irrespective of when the FL was learnt? Or depending on how proficiently balanced people are in their languages? These are relevant lines of inquiry because, recall, the *reduced emotionality* account posits reduced emotional reactions in FL compared with NL. However, emotional responses in FL and NL have been reported to be modulated by age of acquisition [24]. As such, it would be expected that with the NL and FL being closer in age of acquisition, the purported difference in emotional reaction would be reduced and in turn, emotionality should not differentially affect reasoning in NL or FL. Similarly, the *increased-deliberation* account poses that the FLe stems from increased processing costs in the FL, leading to reduced cognitive fluency, which in turn promotes increased deliberation. Under this logic, as the gap in proficiency between languages narrows, there would not be an incurred processing cost in the FL and hence, no increase in deliberative processing in the FL. In fact, Costa et al. [17] performed a post hoc analysis splitting their participants into lower and higher proficiency and found that the number of utilitarian choices in the aforementioned footbridge dilemma was higher for those participants with lower proficiency than in those with higher proficiency.

A study that set out to focus specifically on aspects of the bilingual experience that could modulate the FLe on moral decision making, was recently published by Wong and Ng [32]. They assessed the impact of age of acquisition and language dominance on responses to a series of personal and impersonal moral dilemmas, in Chinese-English young adult early-bilinguals. Results of their study showed no differences in the rate of utilitarian choices when participants performed in their NL or their FL. However, a FLe was crucially observed when language dominance was taken into account: the more dominant participants were in the language they were tested in, the larger difference in their moral judgements of personal versus impersonal dilemmas. Finally, Mækelæ and Pfuhl [33] using a battery of reasoning tasks (including the CRT, base-rate neglect, ratio bias, and a probability matching task) compared the performance of young-adult participants when presented either with their first language, their foreign language, or in a language switching condition. Results of their study showed no differences in performance between the three conditions and thus the authors concluded that deliberation is not increased when using a second language.

To expand on previous findings, the present study aims to test the FLe on logical reasoning in an older population. Age-related declines on certain aspects of cognitive functioning in healthy adults have been well documented in the literature (for discussions see e.g., [34,35,36]). In particular, cognitive abilities related to fluid intelligence (e.g., memory, processing speed, reasoning) have been reported to decline more than those cognitive abilities related to crystallised intelligence (e.g., verbal ability) [35]. A number of accounts have been offered to explain such changes. From a neurobiological point of view, for example, one possibility appeals to frontal networks of the brain being more sensitive to ageing and thus impacting related cognitive abilities (for a review see e.g., [37]). Reasoning is an ability that relies on frontal brain networks and therefore its study on an older population can aid our understanding of cognitive changes in older adulthood.

In addition, and of relevance for the present study, within the framework of dual-processing it has been proposed that deliberative processing (related to System 2) is more likely to decline with age than is affective processing (related to System 1) (for a review see [38]). Based on that observation, studying those populations who are not expected to have a ceiling level on deliberative processing, in this case, older adults, can be more informative to capture an effect. Interestingly, a parallel line of reasoning is found in research on the impact of bi/multilingualism on cognition, and in particular on executive functioning (we thank an anonymous reviewer for raising this point). In that area of research, it has been suggested that certain cognitive effects of bilingualism are more likely to emerge in older adults than in younger adults (see e.g., [39,40]) due to young adults performing at ceiling (but cf. [41,42]). From that perspective, effects of language experience thus seem more likely to be observed in older adults. Although reliance on young adults is a common feature of modern experimental psychology, looking at young populations only is also problematic as it prevents generalizability of findings. Yet, as we have seen, the study of FLe and in particular, in relation to deliberative processing in older adults has been under researched as a large proportion of studies have focused on young adults.

In addition, given that most work on FLe has focused on testing the reduced-emotionality account, the present work sheds light on inconsistent previous findings on performance on CRT (related to increased-deliberation) in the context of the FLe. Furthermore, we take into account bilingualism-related factors (FL proficiency). Our hypotheses are thus based on the FLe in the context of the *increased-deliberation* account, and we reasoned that *increased-deliberation* could be tested in two ways as we outline below.

If thinking in a FL reduces cognitive fluency, and if reduced cognitive fluency engages in deliberative and analytical thinking, our predictions are two-fold: first, we hypothesize that participants performing an analytical task (CRT) in their FL would have a higher rate of correct responses than those performing in their NL. Second, if the cognitive demands imposed by thinking in a FL are reduced at higher levels of proficiency, we predict an inverse relationship between proficiency and performance such that those participants performing the task in the FL shall exhibit better performance at lower levels of proficiency. Finally, we also studied the effects of the demographic factors age and level of education. Regarding age, it has been reported that older adults, compared with younger adults, tended to provide a higher relative proportion of intuitive errors on the CRT 3-item version, whilst an opposite pattern (i.e., younger adults provide a higher proportion of intuitive errors compared with older adults) was reported in a longer 7-item version of the CRT [43]. In a similar vein, Stieger and Reips [44] performed analyses on each CRT item separately and reported that the lower the age, the higher the accuracy rate on item 1 of the classical 3-item version of the CRT. We also account for the effects of education, as it has been reported that higher education is a strong predictor of higher performance on the classic version of the CRT [44].

## 2. Method

### 2.1. Study Population

In this study, we used data collected as part of the project *“Successful aging”,* a study in Umeå, Sweden. The study received ethical approval from the Regional Ethics Committee at Umeå University and all participants gave informed consent. Data were collected over two test waves (T1 and T2), about 3 years apart. For the purpose of the present study, data collected at T1 were used because the CRT was included in the test battery only at that time point. Participants were recruited through advertisement in local newspapers, and through various pensioners and language associations. To be included in the study, participants had to be within 50–75 years old and to consider themselves to be either: (1) bilinguals who used both Swedish and Finnish regularly, or (2) bilinguals who used both Swedish and English regularly, or (3) had none or minor knowledge in any other language than Swedish. All participants were living in Sweden at the time of testing. Participants attended the test locations to take part in two testing sessions at T1, about 1 week apart. Each session lasted approximately 2 h and focused on cognitive assessments using computer-based tasks. In the last part of the second session, a number of paper and pencil tests were also administered, including the CRT (see measures below). Between sessions, participants filled in a number of questionnaires at home (e.g., language questionnaire). For the present purposes, to test the FLe on logical reasoning (CRT performance) in an older population, we identified 152 participants who had complete data on the CRT.

Language and background information was used to categorize participants into either the NL or the FL group. Since all participants performed the tasks in Swedish, information from the language questionnaire (LSBQ, see measures section below) was employed to determine whether Swedish was their NL or their FL and thus to allocate group membership. We operationalised NL as the language acquired from birth (i.e., age of acquisition, AoA = 0), without simultaneous acquisition of two or more languages. We first used place of birth and AoA to categorise. In cases of missing AoA, group membership was determined based on their place of birth and language use in the first years of life (infancy), or place of birth and whether they reported knowledge of one language only. Therefore, participants categorised in the NL group were those born in Sweden and had Swedish as their native language, or those born in Sweden and used Swedish mainly in their first years of life, or those born in Sweden and reported knowledge of Swedish only. Participants categorised in the FL group were those born outside Sweden and had a native language other than Swedish or those born outside Sweden and used mainly a language other than Swedish in the first years of life (Table 1).

Based on the abovementioned criteria, 23 participants were excluded from analyses as follows: two participants with a native language other than Swedish but who had been born in Sweden. Similarly, a participant who learnt Swedish from birth but was born outside Sweden was also excluded from analysis as were 3 participants who reported simultaneous acquisition (AoA = 0) of two or more languages, and one participant who did not report an AoA = 0 in any of the languages they listed. One further participant whose native language was a Swedish dialect but lived in Sweden from birth was also excluded as were participants with incomplete data on the background information to determine group allocation using the abovementioned criteria (*n* = 15). Of the remaining 129 participants, *n* = 84 were categorised in the NL group and *n* = 45 in the FL group.

Within the FL group, most participants were born in Finland (*n* = 42), one in England (*n* = 1), one in Russia (*n* = 1), and one participant did not specify the place but indicated that was not born in Sweden. The language acquired from birth (i.e., AoA = 0, here considered their native language) was Finnish for the majority of participants (*n* = 44), and English for one of the participants in this group. The mean age at which they moved to Sweden was 19.2 (*SD* = 9.58), and their mean AoA of Swedish was 14.2 (*SD* = 9.29). Likely because of the time living in Sweden, a number of participants in this group (*n* = 7) had listed Swedish as their L1 and in terms of proficiency, *n* = 15 had mean self-ratings of Swedish proficiency higher than their first acquired language (here considered their NL), however, they were categorised within the FL group following order and age of acquisition as outlined above (please see Appendix A). Self-rated proficiency in Swedish and use of Swedish at different stages of life is shown in Table 1 and additional language background information is provided in (Appendix A).

### 2.2. Measures

Cognitive reflection test (CRT). At the second testing session at T1, participants performed a 6-item paper and pencil version of the CRT [45] in Swedish. A sample problem from the original test [16] is shown below:

A bat and a ball cost $1.10 in total. The bat costs $1.00 more than the ball.

How much does the ball cost?

Answer: ___cents

Each problem was presented on a separate page. The test was self-administered, and participants solved one problem at a time. Participants were instructed to not go back to any previous problem once they had responded and moved forward. A research assistant conducting the testing session ensured that the protocol was followed accordingly. Each problem was open-ended, and the responses were coded as either correct or incorrect. For each problem, participants also rated their degree of certainty (0 to 100 %) and on a continuous scale indicated whether they considered their response to be completely intuitive (scored as 1) or completely analytical (scored as 11). Participants were also asked to respond whether they had seen any of the problems before. Different randomization orders were used (6 versions), and the maximum time allowed to complete the CRT was 9 min. The sum score, based on the number of correct responses, was used as a proxy of deliberative processing.

Raven’s advanced progressive matrices (APM) test — short version. A short paper version of the Raven’s APM Test [46] was administered as part of the second testing session. The score consisted of the number of correct responses out of 12 items.

Self-rated language proficiency. As part of a Swedish adapted version of the “Language and Social Background Questionnaire” (LSBQ, [47]), participants self-rated their proficiency to speak, understand, read, and write in Swedish and other languages. Self-ratings were on a scale from “no proficiency” (0) to “high proficiency” (10) on each domain. For this study’s purpose, a composite mean score of the four self-rated proficiency items in Swedish was also calculated (Table 1). Information about use of Swedish at different stages in life (infancy, pre-school age, primary school age, high school age, and college/university age) with a scale ranging from “always Swedish” (0) to “always non-Swedish” (10) was also gathered. Additional language information for each group is provided in Appendix A.

Swedish synonym test (SRB-test). This task [48] was also administered as part of the second testing session. The task consists of 30 items, in a multiple-choice format, where participants are asked to select the correct synonym word out of five alternatives. The test was developed to measure vocabulary knowledge in Swedish, and the maximum time to solve the task was seven minutes.

Covariates. Age and years of formal education were used as covariates in the analyses.

### 2.3. Statistical Analyses

Tests of normality (Kolmogorov–Smirnov) as well as Skewness and Kurtosis values are presented in Appendix B. As can be seen in Table A3, tests of normality indicated that except for the SRB and APM tests in the FL group, all the other variables involving continuous scales deviated significantly from normality. Therefore, differences between the FL and NL groups were analysed using non-parametric Mann–Whitney U tests for the continuous variables as well as for the analyses involving ordinal scales (i.e., self-rated Swedish proficiency and Swedish language use at different stages in life). However, to be able to adjust for covariates, we also performed additional analyses using one-way analysis of covariance (ANCOVA). Chi-square tests were employed for categorical variables (gender) and for frequency data (to test for associations between the rate of correctly answered problems in the CRT and language groups). Finally, to investigate if lower levels of proficiency in the FL group were associated with higher scores in the CRT, we performed non-parametric zero-order correlations as well as Pearson’s partial correlations to adjust for covariates. Statistical analyses were performed using IBM SPSS ^®^ v. 28.

## 3. Results

Table 1 shows demographic information and language proficiency scores. There were no significant differences between the two groups with regards to age (*U =* 1501.50, *z* = −1.93, *p =* 0.054), gender distribution (*χ*^2^(*n* = 129) = 1.09, *p* = 0.296) and years of education (*U* = 1526.50, *z* = −1.33, *p* = 0.185). The groups were also comparable on the Raven’s APM short form (*U* = 1884.50, *z* = −0.027, *p* = 0.978).

As expected, the groups differed on their use of Swedish at different stages in life (all *p*s < 0.001). The NL group self-rated their proficiency to speak Swedish (*Mdn* = 9.0) significantly higher than the FL group (*Mdn* = 8.0; *U =* 1437.00, *z* = −2.22, *p* = 0.026). Also self-rated proficiency to write was higher in the NL group (*Mdn* = 9.0) than in the FL group (*Mdn* = 8.0; *U =* 1299.00, *z* = −2.78, *p* = 0.005) but the two groups did not significantly differ on their self-rated abilities to read (*Mdn*_NL_ = 9.0, *Mdn*_FL_ = 9.0; *U =* 1673.50, *z* = −1.03, *p* = 0.302) and understand Swedish (*Mdn*_NL_ = 9.0, *Mdn*_FL_ = 9.0; *U =* 1717.50, *z* = −0.794, *p* = 0.427). The overall composite Swedish mean score was significantly different between the NL (*Mdn* = 9.0) and the FL (*Mdn* = 8.8) groups (*U =* 1421.50, *z* = −2.28, *p* = 0.023). There was also a significant difference between the groups for the Swedish synonym test (SRB) in that the NL group performed better than the FL group (*Mdn* = 25.5 and *Mdn* = 21.0, respectively; *U =* 908.00, *z* = −4.87, *p* < 0.001).

Regarding our first prediction, namely, that the FL group would perform better compared with the NL group on the CRT, first we compared the overall accuracy in the CRT between the groups. There was no significant difference in performance between the NL group (*Mdn* = 1.00, *M* = 1.89, *SD* = 1.63) and the FL group (*Mdn* = 1.00, *M* = 1.67, *SD* = 1.62; *U =* 1705.50, *z* = −0.942, *p* = 0.346). In addition, since the groups did not differ on the covariates [49], namely, the variables age and years of education, we used ANCOVA to adjust for these two covariates. This showed that while both covariates were related to participants’ performance on the CRT (age: *F*(1, 121) = 6.01, *p* = 0.016, *η_p_^2^* = 0.047; years of education: *F*(1, 121) = 3.99, *p* = 0.048, *η_p_^2^* = 0.032), the group effect remained not significant even after controlling for these two covariates (estimated marginal means after controlling for covariates: *M*_NL_ = 2.00, *SE* = 0.18; *M*_FL_ = 1.57, *SE* = 0.24; *F*(1, 121) = 2.06, *p* = 0.154, *η_p_^2^*= 0.017). The parameter estimates for the covariates were for age *b* = −0.063, 95% CI [−0.1132, −0.0121] indicating that CRT accuracy decreased with age overall in the sample, whereas for education, more years of education were associated with better CRT performance overall *b* = 0.059, 95% CI [0.0005, 0.1177] (Please see Appendix A for additional analyses).

Table 2 displays the distribution (in percent) across the six CRT items between the NL and FL groups. There was no significant association between the proportion of correctly answered problems and language group, *χ^2^(n* = 129) = 3.49, *p* = 0.745 (Fisher’s exact *p* = 0.783).

Table 3 displays zero-order correlations between the variables within the FL group. There was a relatively strong association between years of education and CRT performance, even after removal of two extreme outliers (28 and 40 years of education, respectively) *r*_s_(42) = 0.37, *p* = 0.015, 95% CI [0.0688, 0.6139]. However, this association did not reach statistical significance after correcting for multiple comparisons. Age was negatively associated with all measures of Swedish proficiency: the SRB, each of the self-rated sub-domains, and the Swedish mean composite (Table 3), indicating that the older the participants, the lower their Swedish proficiency. After correcting for multiple comparisons, the associations between age and each of the self-rating measures and Swedish mean composite score remained significant, but not the association between age and SRB. In addition, there were strong positive correlations between the Swedish self-rating measures and the objective measure of Swedish, namely, the SRB (Swedish synonym test), which remained after correcting for multiple comparisons (Table 3).

Regarding our second study prediction, namely that participants performing the task in the FL would exhibit better performance at lower levels of proficiency, the correlations between each of the four self-rated proficiency sub-domains and CRT performance (accuracy) in the FL group followed instead a positive direction, however, none reached statistical significance (*p*s ≥ 0.071, Table 3). Controlling for age and years of education using partial correlations only changed the direction of the association between CRT performance and Swedish speaking and writing to negative, but in all four correlations the associations remained non-significant (*p*s ≥ 0.632). The association between the Swedish mean composite score and CRT accuracy was not statistically significant either (*r_s_*(45) = 0.28, *p* = 0.060, 95% CI [−0.0203, 0.5388]). A partial correlation, controlling for age and education yielded a negative association between CRT accuracy and Swedish mean composite score, but still did not reach statistical significance *r*(40) = −0.002, *p* = 0.991, BCa 95% CI [−0.3107, 0.2781]. The relationship between performance on SRB test and CRT accuracy followed a positive direction, however it was not statistically significant either *r_s_*(45) = 0.23, *p* = 0.128, 95% CI [−0.0769, 0.4973], and controlling for age and education in a partial correlation did not change this result *r*(40) = 0.01, *p* = 0.966, BCa 95% CI [−0.4035, 0.4401].

## 4. Discussion

The present study aimed at testing the foreign language effect (FLe) on cognitive reflection (in a logical reasoning task) in older adults. In particular, based on the *increased-deliberation* account, we explored whether performance on the cognitive reflection test (CRT) would differ depending on the language (foreign vs. native) as conflicting findings have been reported in the literature. We also tested if FL proficiency would be inversely associated with performance on the CRT when accounting for age and years of education.

### 4.1. Testing the FLe in Deliberative Reasoning

We first hypothesized that participants performing the task in their FL would outperform those performing in their NL. We did not find evidence for this hypothesis, as both groups performed similarly, and importantly, both groups were also similar in years of education, age, and performance on the short version of the APM (Raven’s) test. Our first set of findings thus fails to support a differential effect of logical reasoning modulated by NL or FL processing. These results are in contrast with those reported by Costa et al. [17] who found that participants using their FL outperformed participants using their NL on a subset of participants who underwent the 3-item version of the CRT (young adults). Of note, as mentioned in the introduction, the study by Costa et al. [17] focused on moral decision making and presented the superior CRT performance in the FL group as evidence to support the claim that the FL group had good understanding of the problems. In addition, the reporting of the CRT results included only the percentage of “participants providing at least one correct answer out of three” ([17], p. 5). Therefore, we cannot ascertain whether the association between the number of correctly answered problems by language group was followed up with statistical tests or remained at the descriptive level. Our own results instead are in line with Costa et al. [5] and those of Mækelæ and Pfuhl [33], who did not find differences in CRT performance between participants performing in the NL or the FL in young adults. Our findings here extend to a population of older adults.

In the present study, the NL group scored higher than the FL group on the Swedish synonym test. The NL group also self-rated their expressive abilities (speaking and writing) higher than the FL group, but the FL did not differ from the NL in the two self-rated measures of Swedish receptive abilities (comprehension and reading). On the one hand, this can be a proxy of good understanding of the problems, but on the other hand, one possible interpretation could be that the FL group had reached such a high level of proficiency in receptive abilities in their FL that the FLe would not be expressed as it did not generate enough cognitive disfluency when reading in the FL.

While there were no group differences in performance in the CRT, results from the ANCOVA showed that both age and years of education predicted performance on the CRT, with education having a smaller effect size. More specifically, age was inversely related to performance and higher education was predictive of better performance. The reason why we observed an age-related decline in deliberative performance is in line with proposals from the literature of judging and decision making that point to age-related declines particularly on processes that engage deliberative decision-making [50]. For example, Finucane and Gullion [51] reported a reduced proportion of correct answers for old-older and young-older adults compared with younger adults in a number of decision-making tasks, including a 6-item version of the CRT. Peters et al. [38] propose that deliberative processing is likely to decrease with age, whilst affective processing should remain more stable over time. The explanation of this is linked to age-related decline in certain frontal executive functions that are in turn associated with deliberative processing [50], for example, Stupple et al. [52] showed that working memory was crucial for successful performance on the CRT (but cf. [53]).

Although it is beyond the scope of this study, the abovementioned finding in itself is interesting as it calls for a need to expand our understanding of possible changes in deliberative decision-making in later adulthood, which can potentially impact everyday life decisions (for a review on dual-process reasoning in decision making in ageing see Peters et al. [38]). The finding that years of education was positively associated with performance on the CRT overall, was in line with previous studies in the literature that have indeed revealed that level of education was the strongest predictor on performance in the CRT ([44], see also [33]).

Importantly, in light of the scope of our study, we were only able to test the *increased-deliberation* account, given that the task considered here, namely the CRT, is not expected to elicit an emotional reaction. As such, our results do not allow us to draw any conclusions about the plausibility of the *reduced emotionality* account.

### 4.2. The Effects of Proficiency and Demographic Factors in the FL Group

For the analyses focusing on the group performing in the FL, we hypothesized that if thinking in a FL reduces cognitive fluency, and reduced cognitive fluency leads to increased deliberation, an inverse relationship between FL proficiency and performance in the CRT would be observed, when accounting for background factors (age and years of education). Our findings did not yield a significant relationship between CRT and any of the measures of Swedish proficiency, namely self-ratings of Swedish proficiency and performance on the SRB (Swedish synonym test).

A potential caveat of our study is that participants performing the task in their FL were living in the FL environment, and therefore their FL might be their dominant language, thus reducing the purported cognitive disfluency that thinking in a FL may generate. Language dominance might be a relevant variable to further explore in the context of deliberative processing, as it has been shown to affect moral decision making [32]. It is also possible that not only language dominance but also the linguistic context itself (NL or FL environment) plays a role on whether the FLe would exhibit. Another limitation of our study is that the nature of the cohort study from which our data originates did not allow us to have a full cross-over design by language of administration and to randomly allocate participants to conditions. We tried to alleviate this caveat by controlling for relevant variables in our analyses, however, as we outline below, further research is needed to explore aspects of deliberative processing in later adulthood using a fully crossed design whereby participants are assigned to FL and NL conditions in larger samples.

### 4.3. Future Directions

Further research on older adults is needed to explore different populations and linguistic backgrounds to aid our understanding of the generalizability (or lack thereof) of the FLe in deliberative processing in later life, given how many people are faced with making decisions in their FL. In this study, most participants in the FL group had Finnish as their native language. In a previous study [54] it was found that bilinguals who speak linguistically similar languages such as Swedish and English, which both belong to the Indo-European family and the Germanic branch, performed better than monolinguals in measures of both episodic recall and letter fluency. However, bilinguals who spoke languages such as Swedish and Finnish (Uralic language family) were not found to perform significantly better than monolinguals. In relation to the FLe, a recent study by Dylman and Champoux-Larsson [55] found a FLe in both the Asian disease problem (risk aversion) and the footbridge problem (moral dilemma) in Swedish-French bilinguals but not in Swedish-English bilinguals. In two further experiments using the footbridge problem no FLe was found for either Swedish-Norwegian or for Norwegian-Swedish bilinguals. The authors concluded that the FLe in risk aversion and morality is affected by the cultural influence of the FL and by language (dis)similarity. Thus, future studies should aim to investigate if there is an influence of linguistic distance also on FLe in deliberative processing, which lacks in the literature at present.

More generally speaking, findings supporting the reality of FLe have been consistently reported, most recently with a meta-analytical study [56] showing that using a foreign language made participants more willing to accept harm in order to maximise outcomes and reduced their risk aversion. At the same time, the underlying mechanisms, robustness, and implications of FLe are still partly unclear and as we discuss in this paper, there is still ample room for development within research on the FLe.

This paper sought to correct a significant limitation of research on FLe, namely its reliance on a specific population (young adults) and here we tested the effect in an older population. However, this paper also offers a plea for more theoretical and empirical work on the topic of FLe. For example, as the very name of FLe suggests, this line of research has focused on a specific subset of bi- and multilinguals. Researchers on FLe use the terms “native” and “foreign” because they have focused usually on sequential bilinguals who have acquired another language, most often in a classroom context. But it is not the case that all bi- and multilinguals using and communicating in two or more languages daily have learnt languages in a classroom context. Interestingly, recent research by Miozzo et al. [57] suggests that language effects on decision-making and moral judgments are not restricted to foreign languages and that the contexts in which bilinguals use a language (native, regional, or foreign) can affect their decisions. We welcome further work to test the generalizability of these effects.

Moreover, precisely as FLe concerns bi- and multilingual populations, it should come as no surprise that the debate around FLe shares some challenges with broader discussions around the effects of bi- and multilingualism on cognition (for discussions see for example [42], [58]). Such challenges include the consideration of aspects related to between-group variability (e.g., immigration status, general cognitive ability), linguistic experience (e.g., age and modality of acquisition, language proficiency, language dominance, immersion, linguistic environment, number of languages spoken, linguistic distance) and methodological aspects (e.g., operationalization of variables, sample sizes, tasks employed). If significant progress is to be made, better study designs, bigger data, more focus on individual differences, and more longitudinal data are needed as well as including new avenues in research of the FLe that have been unexplored (for a discussion on the latter point, see for example [31]).

## 5. Conclusions

Taken together, our results do not provide evidence that thinking in a FL increases deliberation in a group of older adults performing a reasoning task that does not involve an emotional component.

## Figures and Tables

**Table 1:** Participant characteristics.

	All *n* = 129	NL *n* = 84	FL *n* = 45
Age, *M* (*SD*)	65.1 (5.7)	66.0 (5.1)	63.5 (6.4)
Female %	65.1	61.9	71.1
Education, years, *M* (*SD*)	13.3 (4.8)	13.4 (4.3)	13.3 (5.7)
Swedish proficiency, *M* (*SD*):			
Speaking	-	8.6 (1.7)	8.0 (1.7) *
Understanding	-	9.0 (1.2)	8.9 (1.2)
Reading	-	9.1 (1.1)	8.8 (1.7)
Writing	-	8.9 (1.5)	8.1 (1.9) **
Mean composite score Swedish	-	8.9 (1.3)	8.5 (1.3) *
Use of Swedish, *M (SD)*:			
Infancy	-	0.1 (0.5)	10.0 (0.2) ***
Pre-school age	-	0.2 (0.5)	9.6 (1.1) ***
Primary school age	-	1.6 (1.9)	8.8 (2.1) ***
High school age	-	2.2 (2.4)	8.0 (2.6) ***
College/university age	-	2.6 (2.7)	5.4 (3.1) ***
SRB	23.3 (4.7)	24.9 (2.9)	20.2 (5.7) ***
APM Short form	4.6 (2.6)	4.6 (2.4)	4.7 (3.0)

NL = Native language group. FL = Foreign language group. * *p* < 0.05; ** *p* < 0.01; *** *p* < 0.001. Self-rated Swedish proficiency scale range from 0 “no proficiency” to 10 “high proficiency”. Use of Swedish scale range from 0 “always Swedish” to 10 “always non-Swedish”. SRB = Swedish synonym test. APM short form = Raven’s advanced progressive matrices short form. Mean composite score in Swedish: average of the four self-rated proficiency sub-domains. Missing values in the sample: In the NL-group years of education (*n =* 3), the four self-rated Swedish proficiency scores and mean composite score (*n* =1), use of Swedish in infancy, high school, and university age (*n =* 7), use of Swedish in pre-school (*n =* 6), and use of Swedish in primary school age (*n =* 5). In the FL-group: years of education (*n =* 1), Swedish writing proficiency (*n =* 1), use of Swedish in infancy and pre-school age (*n =* 1), use of Swedish in primary school age (*n =* 3), and use of Swedish in high school and university age (*n =* 2).

**Table 2:** Percentage of participants within each language group across the six tasks in the CRT.

Number Of Correctly Responded CRT Tasks	NL(%)	FL(%)
0	17	22
1	35	36
2	23	24
3	11	4
4	6	2
5	4	7
6	6	4

NL = Native language group, FL = Foreign language group.

**Table 3:** Spearman’s correlations among the variables used in the study within the foreign language group (FL).

	1	2	3	4	5	6	7	8	9
1. CRT	-								
2. Age	−0.27	-							
3. Edu	0.43 **	−0.27	-						
4. Swedish speaking	0.17	−0.55 ***	0.30 *	-					
5. Swedish understanding	0.21	−0.56 ***	0.23	0.68 ***	-				
6. Swedish reading	0.27	−0.46 ***	0.30 *	0.54 ***	0.80 ***	-			
7. Swedish writing	0.18	−0.49 ***	0.48 ***	0.72 ***	0.64 ***	0.57 ***	-		
8. Mean composite self-rated Swedish proficiency	0.28	−0.59 ***	0.46 **	0.83 ***	0.82 ***	0.85 ***	0.81 ***		
9. SRB	0.23	−0.32 *	0.29	0.60 ***	0.58 ***	0.47 **	0.47 **	0.58 ***	-

CRT = cognitive reflection test. Edu = years of formal education. SRB = Swedish synonym test. * *p* < 0.05, ** *p* < 0.01, *** *p* < 0.001. Self-rated Swedish proficiency scale range from 0 “no proficiency” to 10 “high proficiency”. Mean composite score in Swedish: average of the four self-rated proficiency sub-domains. Correlations based on *n* = 45, except for those involving years of education (*n* = 44), Swedish writing (*n* = 44), and the correlation between those two variables (*n* = 43) due to a missing value in each of them. Values in bold remain significant after Bonferroni adjusted alpha (0.05/36) = 0.0014.

## Appendix A

### Appendix A.1. Additional Language Background Information

#### Appendix A.1.1. NL Group

Of the 84 participants in this group, 15 participants did not report any knowledge in a language other than Swedish (i.e., did not list any other language at all or had ratings of 0s in proficiency in any other language). Further 49 participants reported knowledge of one other language in addition to their native language (Swedish), 15 participants reported knowledge of two other languages besides Swedish, and 5 participants reported knowledge of three languages besides Swedish. The reported knowledge on the listed languages varied in levels of proficiency. Table A1 shows the average ratings of Swedish and the means of additional languages with ratings above 0 in any sub-domain.

**Table A1:** Participants’ language background information for the native language (NL) group.

	Mean Composite Score	*n*^1^
Swedish proficiency, *M* (*SD*)	8.9 (1.3)	83
Additional language 1 proficiency, *M* (*SD*)	5.3 (2.0)	69
Additional language 2 proficiency, *M* (*SD*)	2.7 (2.0)	20
Additional language 3 proficiency, *M* (*SD*)	1.5 (0.5)	5

Mean composite scores: average of the four self-rated proficiency sub-domains (speaking, understanding, reading, writing). Self-rated proficiency scale range from 0 “no proficiency” to 10 “high proficiency”. One missing datapoint on Swedish proficiency. ^1^ Means calculated on languages rated with any number above ‘0’ in any of the language sub-domains.

#### Appendix A.1.2. FL Group

Out of the 45 participants in this group, 20 reported knowledge both in Swedish (the language in which they performed the task) and in their native language (Finnish, or English), a further 18 participants within this group reported knowledge of one additional language in addition to Swedish and their native language, and 7 further participants reported knowledge of two additional languages besides Swedish and their native language. Similarly to the NL, levels of proficiency in the ratings varied. The mean ratings of each language with ratings above 0 in any sub-domain are shown on Table A2.

**Table A2:** Participants’ language background information for the foreign language (FL) group.

	Mean Composite Score	*n*^1^
Swedish proficiency, *M* (*SD*)	8.5 (1.3)	45
L1 proficiency, *M* (*SD*)	8.6 (1.5)	44
Additional language 1 proficiency, *M* (*SD*)	5.9 (2.6)	25
Additional language 2 proficiency, *M* (*SD*)	3.7 (1.7)	7

Mean composite scores: average of the four self-rated proficiency sub-domains (speaking, understanding, reading, writing). Self-rated proficiency scale range from 0 “no proficiency” to 10 “high proficiency”. L1 proficiency refers to the ‘native language’ as operationalized in this study i.e., based on order of acquisition: Finnish or English (one participant). One missing datapoint on Finnish proficiency. ^1^ Means calculated on languages rated with above ‘0’ in any of the language sub-domains.

### Appendix A.2. Additional Analyses

Due to the nature of the data of this study and as pointed out by a reviewer, a potential caveat concerns the possible effects of bilingual/monolingual status and/or language proficiency in the FL. Recall that the NL group included participants who were monolinguals in Swedish or did not report proficiency in other languages besides Swedish. Similarly, the FL included a few cases of participants who listed Swedish as their L1, or rated Swedish higher than Finnish, yet they were classed in the FL according to our operationalization of native language (based on place of birth and age of acquisition). In an attempt to reduce the potential effect of the abovementioned factors, we conducted additional analyses comparing the NL and the FL groups but excluding participants in the NL group who could be considered functionally ‘monolingual’ (*n* = 15), i.e., those who listed no other languages besides Swedish or had ratings of ‘0’ only in all domains of any other language. All other participants, with any degree of fluency in a language other than Swedish (from minimal to full proficiency, see Table A1 for more details), remained in the analysis. Similarly, for the FL, as mentioned in the method section, 7 participants in this group had listed Swedish as their ‘L1’ in the language questionnaire. In terms of proficiency, 15 participants in this group had a mean overall Swedish proficiency score higher than their native language. Therefore, for these additional analyses, we also excluded participants in the FL group (*n* = 16) whose mean proficiency ratings in Swedish (i.e., the language in which they performed the task) were higher than the mean proficiency ratings in their native language (*n =* 15) and one case which could not be determined due to missing Finnish ratings.

An initial analysis comparing performance on the CRT between the two groups did not show differences on overall CRT accuracy rates: NL group *Mdn* = 1.00, *M* = 1.96, *SD* = 1.66; FL group *Mdn* = 1.00, *M* = 1.90, *SD* = 1.90; *U =* 928.50, *z* = −0.577, *p* = 0.564. Importantly, the groups did not differ on age (*U =* 922.50, *z* = −0.609, *p* = 0.542) or Raven’s score (*U =* 880.50, *z* = −0.940, *p* = 0.347), but unlike the original analysis, the two groups now differed on years of education (NL group *Mdn* = 14.00, *M* = 13.47, *SD* = 4.42; FL group *Mdn* = 12.00, *M* = 12.52, *SD* = 6.15; *U =* 698.00, *z* = −2.192, *p* = 0.028). However, to mirror the original set of analyses, we used ANCOVA to include the two covariates (age and years of education) and results of this analysis only showed an effect of age in the model *F*(1, 92) = 8.29, *p* = 0.005, *η_p_^2^* = 0.083, but in contrast with the original analysis, no effect of education *F*(1, 92) = 2.34, *p* = 0.129, *η_p_^2^* = 0.025, and still no significant effect of group *F* < 1. Similarly, a chi-square analysis did not show a significant association between belonging to either the FL or the NL group and rate of correctly answered tasks on the CRT *χ^2^(n* = 98) = 5.18, *p* = 0.521 (Fisher’s exact *p* = 0.553).

## Appendix B

**Table A3:** Skewness, Kurtosis, and normality tests by group.

Measure	Skewness (*SE*)		Kurtosis (*SE*)		K–S	
	NL	FL	NL	FL	NL	FL
Age	−0.78 (0.26)	−0.67 (0.35)	0.59 (0.52)	−0.53 (0.69)	0.136 ***	0.169 **
Years of education	−0.47 (0.27)	20.72 (0.36)	0.07 (0.53)	11.18 (0.70)	0.103 *	0.221 ***
Swedish proficiency						
Speaking	−1.36 (0.26)	−1.08 (0.35)	1.31 (0.52)	1.59 (0.69)	0.241 ***	0.179 ***
Understanding	−1.28 (0.26)	−1.23 (0.35)	1.32 (0.52)	1.39 (0.69)	0.264 ***	0.248 ***
Reading	−1.50 (0.26)	−2.98 (0.35)	2.07 (0.52)	11.52 (0.69)	0.255 ***	0.259 ***
Writing	−1.68 (0.26)	−1.55 (0.36)	3.02 (0.52)	3.27 (0.70)	0.249 ***	0.235 ***
Mean composite	−1.29 (0.26)	−1.34 (0.35)	1.48 (0.52)	3.58 (0.69)	0.218 ***	0.148 *
Use of Swedish						
Infancy	4.37 (0.27)	−6.63 (0.36)	21.34 (0.54)	44.00 (0.70)	0.518 ***	0.537 ***
Pre-school age	3.82 (0.27)	−2.89 (0.36)	15.62 (0.54)	7.69 (0.70)	0.516 ***	0.503 ***
Primary school age	0.99 (0.27)	−1.73 (0.37)	−0.04 (0.53)	2.09 (0.72)	0.244 ***	0.343 ***
High school age	1.16 (0.27)	−1.15 (0.36)	0.90 (0.54)	0.34 (0.71)	0.202 ***	0.273 ***
College/university age	0.75 (0.27)	0.17 (0.36)	−0.46 (0.54)	−1.25 (0.71)	0.213 ***	0.132
CRT Total accuracy	1.09 (0.26)	1.34 (0.35)	0.60 (0.52)	1.25 (0.69)	0.220 ***	0.241 ***
SRB	−0.83 (0.26)	−0.51 (0.35)	0.74 (0.52)	−0.36 (0.69)	0.141 ***	0.111
APM Short form	0.27 (0.26)	0.36 (0.35)	−0.79 (0.52)	−0.67 (0.69)	0.153 ***	0.125

K–S Kolmogorov–Smirnov Test with Lilliefors correction. *SE* = standard error. NL = native language group, FL = foreign language group. Self-rated Swedish proficiency scale ranges from 0 “no proficiency” to 10 “high proficiency”. Use of Swedish scale ranges from 0 “always Swedish” to 10 “always non-Swedish”. CRT = Cognitive reflection test. SRB = Swedish synonym test. APM short form = Raven’s advanced progressive matrices short form. * *p* < 0.05, ** *p* < 0.01, *** *p* < 0.001.
